# Evaluating Health Education Module on Hand, Food, and Mouth Diseases Among Preschoolers in Malacca, Malaysia

**DOI:** 10.3389/fpubh.2022.811782

**Published:** 2022-03-31

**Authors:** Syazwani Shahar, Hayati Kadir Shahar, Sri Ganesh Muthiah, Kulanthayan K. C. Mani

**Affiliations:** ^1^Department of Community Health, Faculty of Medicine and Health Sciences, Universiti Putra Malaysia, Serdang, Malaysia; ^2^Malaysian Research Institute of Ageing (MyAgeing), Universiti Putra Malaysia, Serdang, Malaysia

**Keywords:** Health Belief Model, Hand, Foot and Mouth Disease, hygiene, infection, children, preschool, six-year-old

## Abstract

This study aims to improve parents' perceptions of susceptibility, severity, benefits, and barriers to children's handwashing practice by utilizing the Health Belief Model. In Alor Gajah, Melaka, a parallel cluster-randomized controlled study was conducted over 26 months. Parents who agreed to participate completed pre-test (t0) questionnaires. Data analysis used IBM SPSS version 25. The descriptive analysis described the baseline data pre-intervention. Chi-square and *T*-test or Mann-Whitney U test for non-parametric analysis assessed baseline data comparability between intervention and control groups. Generalized Estimating Equation (GEE) analyzed between and within-group comparison of the outcomes, and multivariate analysis determined the effectiveness of the intervention with clustered data. The individual participation rate was 86%. Parents who followed up immediately had higher perceived susceptibility, perceived severity, and perceived barriers (*p* < 0.001). Each unit increment in parents' practice score was 0.02-unit higher preschool children's hand hygiene practice score (*p* = 0.045). The intervention effectively improved parents' perceived susceptibility and benefits at immediate follow-up compared to baseline. However, there were no significant intervention effects on parents' perceived severity and barriers and preschool children's handwashing practices. The follow-up time significantly affected each outcome. There were significant covariates as the outcome predictors in this study, besides intervention groups and follow-up time. Parents' knowledge and age of the youngest child were significant predictors of parents' perceived susceptibility, besides parents' knowledge and perceived susceptibility being the predictors of parents' practice score. As a result, parents, teachers, and communities can implement this intervention in other schools with susceptible children.

## Introduction

Since our first case of Hand, Foot, and Mouth Disease (HFMD) in 1997, Malaysia has become an endemic country of this disease (1). HFMD incidence increased more than 150% in 2018 compared to the previous year. On top of that, more than 50,000 people had been affected by this epidemic, and most were children (2). The age-weighted Disability Adjusted Life Years (DALYs) was 96,900 annually (95% CI: 40,600–259,000) in eight high-burden countries in Asia, and the majority were attributed to Years of Life Lost (YLL) (1). It has a mortality rate of 52.3 per 100,000 symptomatic patients, and it can be even higher if Enterovirus 71 (EV71) is the cause (230 per 100,000 cases).

Increased parental awareness of the importance of preschool education for their children and an increase in the number of children requiring early education in Malaysia has resulted in more preschools to meet the demand. Due to space constraints, most classrooms can only hold 20 to 30 students (2). As a result, the congested area creates an environment that promotes the spread of infectious diseases, particularly those transmitted through airborne droplets or contaminated surfaces, such as HFMD.

Preschool children are among those who are susceptible to contracting the disease due to the crowded environment in educational institutions. Although the incidence rate of HFMD was highest among children under the age of five in the literature (1, 3, 4), the HFMD incidence among six-year-old children was still significant and needed to be intervened, according to a modeling study, in which the incidence of HFMD (2003 until 2012) among this age group was 16.5 per 1,000 person-years, a slight reduction compared to 24.3 per 1,000 person-years among children aged five years old (1). Furthermore, a cross-sectional study of HFMD cases reported in China from 2001 to 2007 found a gradual decrease in the proportion of cases among children aged 0–4 years old, with a corresponding shift in the age distribution of HFMD cases among older children aged 5 to 9 years old (4). The proportion of cases in this age group was significantly increased from 17.0% in 2001 to 26.2% in 2007 (*p* < 0.05).

Furthermore, in terms of a lifecycle-based health promotion policy, the age range of 6 to 12 is critical for children to begin adopting health-promoting behaviors, such as hand hygiene (5). The good habits formed may influence these children's lifelong health habits and long-term health into adulthood during this stage. Children are more likely to be affected by adult behaviors at home because they rely on their parents and families. As a result, parents should be included in any intervention to improve children's health behavior, including hand hygiene.

Except for symptomatic treatment, there is no known effective treatment for controlling and preventing HFMD infection. Nonetheless, the monovalent, inactivated EV71 vaccine, used in China since 2008, claims to protect a child from HFMD infection within 10 days of administration (6). However, it is ineffective against other strains and has no long-term effects. No other country has proven its effectiveness in preventing HFMD. As a result, hand hygiene is the most practical, simple, and cost-effective method of preventing and controlling HFMD infection and outbreaks. Handwashing behavior was linked to HFMD infection in a case-control study conducted in China (*OR* = 0.41, 95% CI: 0.19–0.89), and handwashing before meals was a significant protective factor from HFMD (*OR* = 0.3, 95% CI: 0.13–0.70) after controlling for confounders (7). Furthermore, many interventional studies have found that intervening in hand hygiene practices reduces the incidence of HFMD (8, 9).

However, parents' hand hygiene practice is still low despite having a high level of knowledge and attitude, shown by a cross-sectional study conducted in Klang Valley among preschool children's parents (10). Furthermore, two-thirds of parents disagreed that they should wash their hands before touching their children, and three-quarters were unaware of proper handwashing techniques. Again, there was a shortage of interventional studies in Malaysia to examine the effectiveness of health education on hand hygiene, emphasizing the role of parents in improving their children's handwashing habits. In addition to the importance of parents' knowledge and practice of hand hygiene in shaping their children's handwashing behavior, they may also act as symptomatic carriers and transmit the infection to the children in their care if they fail to follow preventive practices (11). Instead of transmitting the disease to the children under their care, they also might develop clinical HFMD due to taking care of infected children. A follow-up study in China reported having children diagnosed with HFMD recently is among the risk factors of HFMD in adults (12). In this case, rather than being the savior for the kids, they will only worsen the condition and further contribute to the HFMD outbreak.

As far as hand hygiene is concerned in Malaysia, there are no proper guidelines to prevent infections, including HFMD. School health nurses provide informal hand hygiene education to preschools and primary schools once a year but without proper guidelines or assessment. Concurrently, *Tunas Doktor Muda*, a preschool-based branch of Young Doctors' Club, has included hand hygiene as part of its curriculum. Preschool teachers conduct individual assessments and evaluations on each module element. The findings, however, have not been made public.

The importance of parents' involvement in determining their children's hand hygiene practices, as well as the escalating incidence of HFMD in Malaysia in recent years without properly guided, effective preventive measures, the lack of interventional study to improve parents' knowledge, attitude, and practice on HFMD and hand hygiene in the educational setting, and the lack of interventional study to improve parents' knowledge, attitude, and practice on HFMD and hand hygiene in the educational setting, are the reasons for this trial. We used a theory to guide us through designing, implementing, and evaluating the intervention. The Health Belief Model was chosen over other theories because it emphasizes modifying factors to the perception and self-efficacy components.

In addition, cues to action are an essential component in mediating individual beliefs. Furthermore, hand hygiene interventions have been effective in healthcare and community settings (13, 14). Effective preventive measures should be developed to break the HFMD transmission chain and reduce the disease's burden in Malaysia, particularly among young children. Preschools should be the safest and most conducive environment outside of the home for children to gain knowledge and experience without being exposed to infectious agents.

### Research Questions

i. Does the health education on hand hygiene and HFMD among parents using Health Belief Model demonstrate an intervention effect overtime on parents' perceived susceptibility, severity, benefits and barriers?
ii. Does the health education on hand hygiene and HFMD among parents using Health Belief Model demonstrate an intervention effect overtime on handwashing practice among their preschool children?

### Research Objectives

#### General Objective

To develop, implement, and evaluate the health education module's effectiveness in improving parents' perceived susceptibility, severity, benefits and barriers and preschool children's handwashing practice using the Health Belief Model.

#### Specific Objectives

i. To develop, implement and validate the health education module on hand hygiene and HFMD using the Health Belief Model.
ii. To compare baseline data regarding sociodemographic status, household characteristics, parents' knowledge, perceived susceptibility, severity, benefits and barriers, and practice of hand hygiene and HFMD, and preschool children's handwashing practice between intervention and intervention control groups.
iii. To compare the hand hygiene and HFMD health education module's effectiveness on parents' perceived susceptibility, severity, benefits and barriers, and preschool children's handwashing practice at baseline, immediate post-intervention and three-month post-intervention between and within the intervention and control groups after controlling for confounders.

## Materials and Methods

### Design and Setting

This 26-month double-blind parallel cluster-randomized controlled trial was undertaken in Alor Gajah, Melaka. The study population included parents of six-year-old preschoolers in Alor Gajah. The primary sampling frame includes preschools visited by the school health team. The secondary sample frame was the preschool parent lists. The sample unit is a parent of a six-year-old preschool student from Alor Gajah.

The sample size was calculated by comparing two proportions and two means (15). The sample size is based on 80% power, 0.05 significance, and a 20% dropout rate. The sample was then adjusted by 1.95, based on an intracluster correlation coefficient (ICC) of 0.05 (16), with 16 clusters averaging 20 children each. The overall sample size was 133 individuals per arm, distributed into 8 clusters of roughly 20 children each school. The study participants were the parents who consented to join this study and filled in the pre-test (t0) questionnaires.

### Randomization, Allocation and Blinding

The school health team provided a list of preschools. Those who refused to conduct the study among their preschool children's parents were removed from the list. Then, using a random sequence generator, 16 pre-schools were chosen at random from a simple random sampling. Eligibility criteria were used to evaluate preschool and preschool students. The investigator was then blinded to the number of blocks by performing a permuted block randomization with different block sizes. The block's dimensions were the same as 2 and 4. Different block intervention groups were assigned to pre-schools.

Allocation concealment was used to avoid selection bias. A sealed, opaque envelope containing each label's assigned intervention group, such as A or B, was used to conceal the randomization. The given group will only be revealed to the investigator after randomization.

This study used a double-blinding method, which meant that neither the participants nor the liaison officers knew which group they were in. This safeguard is in place to avoid ascertainment bias. The researcher could not be blinded while performing the intervention.

### Selection Criteria (Individual and Cluster Level)

Inclusion criteria included all government or non-government preschools that Alor Gajah School Health Team visits (at cluster level) and parents of six-year-old preschool children in Alor Gajah (individual level).

Exclusion criteria include parents of children who were cared for in nurseries or daycare centers (at an individual level), parents who are illiterate in Malay and English language (at an individual level), preschools that were under the same administration as any elementary school (at cluster level). This measure avoids historical effects that will threaten the study's internal validity if there are other interventions that the school administration conducts in parallel with this intervention. Also, preschools who received hand hygiene interventions other than standard care in the past year were excluded (at cluster level).

### Health Education Module

**Figure 1:**
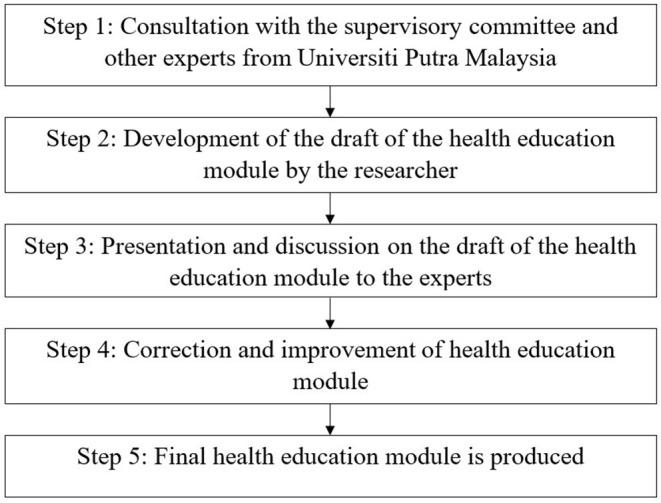
The schematic diagram for the development of the health education module on prevention of Hand, Foot and Mouth Disease.

The Public health experts from Universiti Putra Malaysia (UPM) and the Alor Gajah District Health Office validated the health education module in Malay and English versions. A Public Health Specialist and a Malay translator are among the experts. The flow of a health education module development is depicted in Figure 1.

The application of the Health Belief Model (HBM) components in the development of the module. The recorded health talk and brochure distribution detailed the perceived susceptibility, severity, and benefits. In addition, one of the videos discussed the importance of soap-based handwashing in preventing disease transmission. The perceived barriers were explained by a recorded health talk that listed possible hand hygiene barriers from previous studies and how to overcome them. The cues to action were applied through the distribution of the brochure, with the hope that having the brochure with them at home and reading it multiple times would remind them to wash their hands properly. Based on National Health Services (NHS), the self-efficacy construct in HBM was applied in the module via recorded videos on proper handwashing techniques. The NHS handwashing technique was used in the module because it is more detailed and understandable to the layperson, and it is based on the 13 steps of handwashing.

### Quality Control

The scales were forward-backward translated into Malay by bilingual native speakers fluent in English and two Malay language experts. The consolidated version has minor linguistic and cultural adjustments. Due to the COVID-19 pandemic, 60 preschool parents from Melaka Tengah participated in the module's online pilot testing in May 2020. The intervention group received recorded health talk, videos on handwashing techniques and the importance of handwashing with soap and water, and brochures via preschool teachers as liaison officers. The control group received the same brochures on hand hygiene and HFMD from the liaison officers among the responders. Online questionnaires were used to collect data before and after the intervention. Feedback from audiences was used to measure instrument recognition, acceptability, and target population relevance.

Face validity was assessed by a group of public health graduate students. The questionnaires' substance and format were evaluated. A panel of five experts assessed the questionnaires' content validity (3 from the field of expert, two from other fields). Panelists were asked to rank the items on a three-point ordinal scale for necessity (not necessary, useful but not essential and essential). The content validity ratio ranges from 1 to −1. The higher the score, the more the panel members agree on an item's relevance. The CVR ranges from 0.6 to 1. As a result, everything stayed in the instrument.

The questionnaires' reliability was tested on 30 people who shared the same characteristics as the study participants (parents of preschool children). As measured by Cronbach's alpha, internal consistency was used to assess the scale's reliability.

### Data Collection Process

Respondents were sent an online survey link for the study's baseline data. The intervention package was supplied a week after the baseline data. A liaison officer selected among preschool teachers managed the delivery of intervention packages. The health education program lasted about an hour. A post-intervention survey will be provided after the health intervention (T1). Parents' and students' knowledge, attitude, and hand hygiene practice were reassessed three months later (T2). The control group received the same brochure as the intervention group. The data was collected in the same way as the intervention group: at baseline (T0), immediately after the brochure was distributed (T1), and three months later (T2).

### Data Analysis

Data analysis was done using IBM SPSS version 25. This study used a significance level with a *p*-value of 0.05 and a confidence interval of 95%. For results yielded *p* < 0.05, there is a statistically significant improvement in the parents' perceived susceptibility, severity, benefits and barriers, and preschool children's handwashing practice after a health education module intervention based on the Health Belief Model. The normality testing was carried out to determine the distribution of continuous data. The descriptive analysis described the baseline sociodemographic, household characteristics, parents' knowledge, practice, perceived susceptibility, severity, benefits and barriers on hand hygiene and Hand, Foot and Mouth Disease (HFMD) and preschool children's handwashing practice pre-intervention. Chi-square and *T*-test (or Mann-Whitney U test for non-parametric analysis) were used to assess baseline data comparability between intervention and control groups for categorical and continuous data. Generalized Estimating Equation (GEE) was used to analyze between and within-group comparison of the outcomes. It was also used as multivariate analysis to determine the effectiveness of the intervention with clustered data. GEE was chosen as the bivariate and multivariate analysis as it is best used in correlated data (cluster randomization and repeated measures). It provides an unbiased estimation of population-averaged regression coefficients.

Regarding sensitivity analysis, the missing data were analyzed to determine the pattern, either missing at random (MAR), missing completely at random (MCAR) or missing not at random (MNAR). Next, multiple imputations were used to handle missing data in the case of MCAR. Intention-to-treat (ITT) analysis was used because the trial's objective was to assess an intervention's effectiveness rather than efficacy. It was a community trial meant to assess an intervention's effect in real-world settings. Therefore, the report of the effectiveness of this trial was based on the intention to treat analysis. However, as there were withdrawals from the trials at follow-ups, sensitivity analysis was done to compare ITT results to the per-protocol analysis (PPA), which only analyzed the complete data. The results of PPA for all outcomes are placed in the appendix for further reference.

For ITT analysis, all 134 participants in the intervention and control group who were randomized were analyzed. For PPA, only 126 in the intervention group and 122 in the control group who completed the study were analyzed.

### Ethical Consideration

The Human Ethics Committee of Universiti Putra Malaysia consented to perform this research (UPM). The study was registered with clinicaltrials.gov (Trial number: TCTR20200211003). A fact sheet and informed consent to parents were also prepared and distributed to each participant before the study to ensure understanding and privacy of respondents' details. Permission from respective preschools was also obtained. The principal investigator is trained in Good Clinical Practice (GCP) throughout the research to safeguard participant rights, safety, and well-being.

## Results

### Response Rate

**Figure 2:**
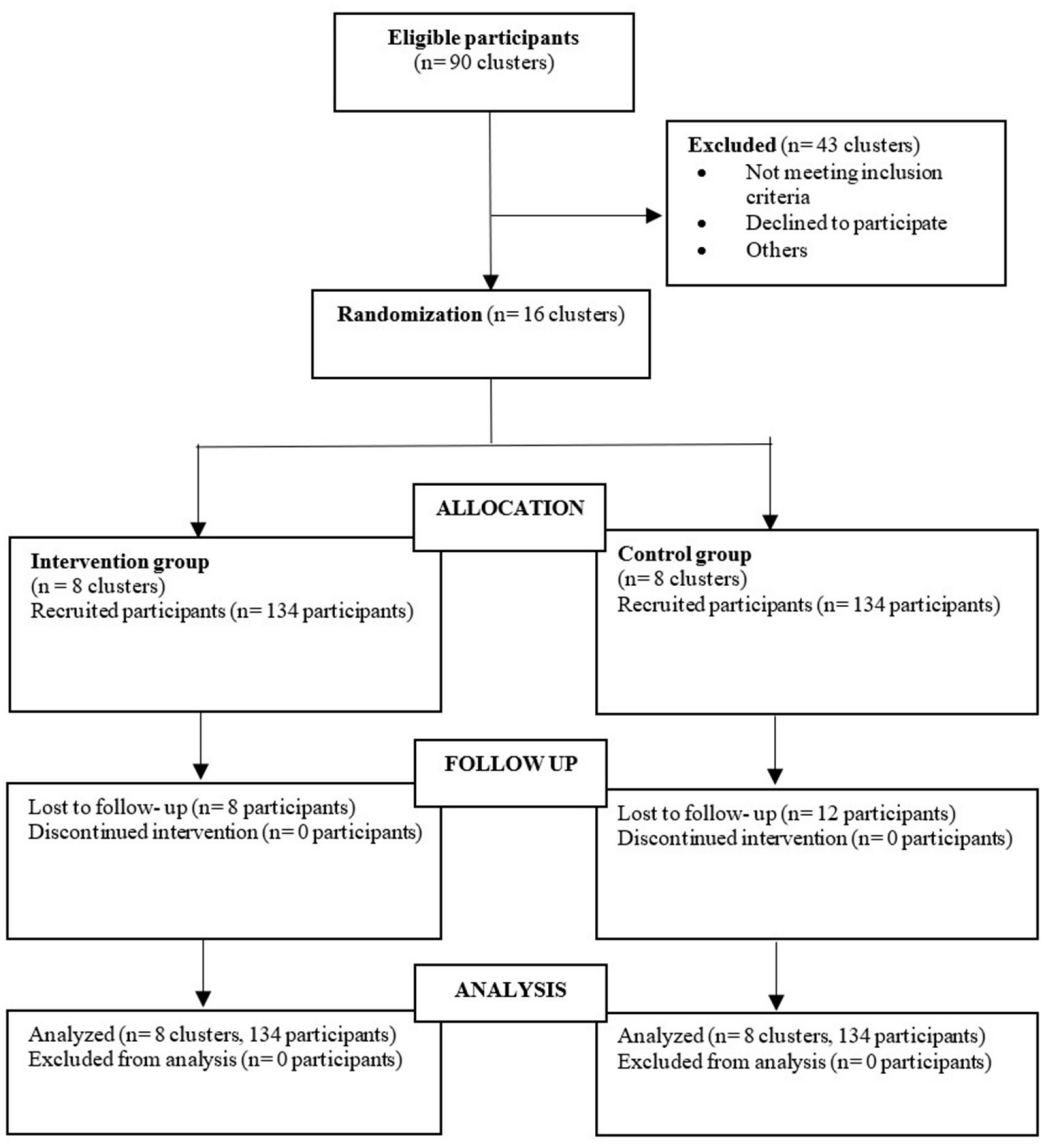
CONSORT flow diagram.

One department involving 20 preschools did not allow the survey out at their preschools (KEMAS preschools) due to the COVID-19 pandemic. The other 23 preschools were not eligible under the same administration as primary schools. Sixteen preschools were randomly selected using a random sequence generator from the remaining 47 preschools. The preschools were randomly allocated into intervention groups. Eight preschools were appointed as intervention and control groups, respectively. A total of 311 students was in the selected preschools (154 students in the intervention group and 157 students in the control group). Out of 311 parents of preschool children, only 268 agreed to participate in the study, making the individual participation rate 86%. It was balanced between the intervention group (*n* = 134, 87.0%) and the control group (*n* = 134, 85.3%). Throughout the study, eight parents (three from the intervention group and five from the control group) defaulted at immediate post-intervention. In comparison, 12 parents (five from the intervention group and seven from the control group) defaulted at third-month post-intervention, making the total attrition rate 7.4%. Figure 2 summarizes the final research flow chart based on the CONSORT statement.

### Missing Data

The missing data were analyzed, giving rise to a total of 3.5% missing data. The missing data came from defaulted cases at T1 and T2 and missing completely at random (MCAR) by Little's test. They were handled using multiple imputations techniques. The predictive mean matching (PMM) method was used for multiple imputations, with five datasets were generated.

### Baseline Comparison of Socio-Demographic, Household Characteristics, Parent Perceptions and Children's Handwashing Practice

Table 1 depicts the comparison of the baseline data on sociodemographic, household characteristics, parent perceptions and children's handwashing practice between intervention and control arms. There were significant differences between intervention and control arms for continuous variables regarding parents' age (*i* = −2.566, *p* = 0.011) and the youngest child's age (*p* = 0.024). Both variables were higher in control groups. The categorical data showed no differences between intervention and control arms across variables except residence location (χ*2* = 48.462, *p* < 0.001). Most of the intervention group participants lived in urban areas, while most control groups lived in suburban areas. Most of the respondents in both intervention and control arms were mothers to preschool children, Malay, who attended primary or secondary school and married. More than half of the preschool children were male.

**Table 1:** Baseline comparison of socio-demographic, household characteristics and parent's perception and children's handwashing practice (*n* = 268).

**Characteristics**	**Intervention**		**Control**		**Independent** ***T*****-test**	
	**Mean**	**SD**	**Mean**	**SD**	**T statistics**	***P*-value**
Age of parents	32.97	5.68	34.66	5.14	−2.566	0.011
Perceived severity	9.05	1.87	8.97	1.75	0.371	0.711
Perceived barriers	11.02	2.54	10.72	2.61	0.972	0.332
**Characteristics**	**Median**	**IQR**	**Median**	**IQR**	**Mann-Whitney U test**	
					***P*** **-value**	
Youngest child age	3.00	3.70	3.15	3.82	0.024	
Perceived susceptibility	12	4	12	5	0.115	
Perceived benefits	12	4	12	4	0.504	
Children's handwashing practice score	15	1	15	2	0.291	
**Characteristics**	**N**	**%**	**N**	**%**	**Chi-square test**	
				**χ**^2^ **statistics**	***P*** **-value**	
**Gender**						
Male Female	70 64	51.9 48.1	74 60	55.2 44.8	0.307	0.579
**Location of residence**						
Urban Suburban Rural	77 39 18	57.8 28.9 13.3	23 66 45	17.2 49.3 33.6	48.462	<0.001
**Relationship**						
Father Mother Others	16 117 1	11.9 87.4 0.7	18 113 3	13.4 84.3 2.2	1.222	0.543
**Ethnicity**						
Malay Non-Malay	127 7	94.8 5.2	130 4	97.0 3.0	0.830	0.362
**Level of Education**						
No formal education Primary/secondary school Certificate/Diploma Degree/Master/PhD	4 63 42 25	3.0 46.7 31.9 18.5	1 67 38 28	0.7 50.0 28.4 20.9	2.398	0.494
**Marital status**						
Married Single Divorced/ Widowed	124 2 8	92.6 1.5 5.9	126 3 5	94.0 2.2 3.7	0.893	0.640
**No of household**						
1 2 3 or more	5 12 117	3.7 8.9 87.4	2 12 120	1.5 9.0 89.6	1.299	0.522
**Sharing room**						
Yes No	122 12	91.1 8.9	124 20	85.1 14.9	2.338	0.126
**No. of people sharing room**						
1 2 3 4 or more Not related	22 43 32 25 12	16.3 32.6 23.7 18.5 8.9	26 37 32 19 20	19.4 27.6 23.9 14.2 14.9	3.753	0.440
**Active smokers**						
Yes No	68 66	51.1 48.9	86 48	64.2 35.8	4.373	0.030
**No of active smokers**						
1 2 3 or more Not related	60 7 2 66	44.4 5.2 1.5 48.9	75 10 1 48	56.0 7.5 0.7 35.8	5.368	0.147

Furthermore, all variables showed non-significant differences between the intervention and control group except living with active smokers (χ^2^ = 4.373, *p* = 0.03). The majority of respondents in the intervention and control groups claimed they lived together with active smokers. However, the discrepancy was big, in which only 51.1% of respondents in the intervention group lived with smokers, compared to 64.2% of them living together with active smokers in the control arm. Most respondents in both groups lived with more than three people in the same household and shared a room with two people. Most of the children who lived with active smokers lived with one smoker in the same environment.

Moreover, the baseline comparison between intervention and control groups on perceived susceptibility, severity, benefits and barriers of parents and handwashing practice of preschool children shows no significant differences. Hence, based on the baseline comparison of the sociodemographic and household characteristics and the outcomes, both trial groups were partially comparable as they were similar in most of the variables, except the age of parents, youngest child age, location of residence and numbers of active smokers.

Concerning attitude subscales, our trial reported the highest score in the perceived barriers and perceived susceptibility constructs. The perceived severity subscale had the lowest total score. The item “I think that HFMD is a very severe disease” received the lowest score out of all items (Mean = 2.84, SD 1.22 for the intervention group vs. Mean = 2.56, SD 1.14 control group).

Also, our study reported an excellent handwashing practice among preschool children as self-reported by their parents (Median = 15, IQR 1 in intervention group vs. Median = 15, IQR 2 in control group), with no significant difference between both groups. This finding can be related to the nature of data collection, which we depend on parents' reporting on their children's handwashing practice.

### Effectiveness of the Module on Perceived Susceptibility, Severity, Benefits, Barriers, and Preschool Children's Handwashing Practice

#### Perceived Susceptibility

The effectiveness of the health education module on parents' perceived susceptibility was analyzed using intention to treat analysis and compared to the per-protocol analysis. The final model's smallest Quasi Likelihood under Independence Model Criterion (QIC) was 2,695. Table 2 depicts the timepoints, total knowledge score, age of youngest children and interaction term between group and time were the significant model effects. Parents who followed- up immediately after the intervention had higher perceived susceptibility compared to baseline (*B* = 0.719, 95% CI: 0.387–1.751, *p* < 0.001), and those who were at third-month post-intervention recorded increment in parents' perceived susceptibility by 0.5 unit (*B* = 0.517, 95% CI: 0.171–0.862, *p* = 0.003).

**Table 2:** Effectiveness of the module on perceived susceptibility, severity, benefits, barriers, and preschool children's handwashing practice.

**Items**	**Variable**	**B^**b**^**	**SE**	**Wald**	**95% CI**		***P*-value**
					**Lower**	**Upper**	
Perceived	Trial group						
Susceptibility^b^	Control^a^						
	Intervention	0.264	0.226	1.367	−0.706	0.378	0.242
	Timepoint						
	Baseline^a^						
	Immediate follow-up 3 months follow-up	0.719 0.517	0.169 0.176	18.035 8.604	0.387 0.171	1.751 0.862	<0.001 0.003
	Level of education						
	Lower education^a^						
	Higher education	0.323	0.203	2.530	0.075	0.722	0.112
	Age of youngest child	−0.116	0.055	4.488	−0.224	−0.009	0.034
	Total knowledge score	0.245	0.012	453.244	0.223	0.268	<0.001
	Trial groups x time point						
	Control x baseline^a^						
	Intervention x immediate follow-up Intervention x 3 months follow-up	0.510 0.426	0.228 0.239	4.980 3.179	0.062 −0.042	0.957 0.894	0.026 0.075
Perceived	Trial group						
Severity^c^	Control^a^						
	Intervention	0.107	0.230	0.219	−0.343	0.558	0.640
	Timepoint						
	Baseline^a^						
	Immediate follow-up 3 months follow-up	0.603 0.575	0.155 0.159	15.172 13.130	0.299 0.264	0.906 0.887	<0.001 <0.001
	Level of education						
	Lower education^a^						
	Higher education	0.223	0.192	1.357	−0.152	0.598	0.244
	Age of youngest child	0.050	0.049	1.053	−0.045	0.145	0.305
	Total knowledge score	0.013	0.014	0.876	−0.014	0.040	0.349
	Trial groups x time point						
	Control x baseline^a^						
	Intervention x immediate follow-up Intervention x 3 months follow-up	0.169 0.075	0.253 0.251	0.447 0.090	−0.327 −0.147	0.665 0.567	0.504 0.765
Perceived	Trial group						
Benefits^d^	Control^a^						
	Intervention	0.024	0.217	0.013	−0.450	0.401	0.911
	Timepoint						
	Baseline^a^						
	Immediate follow-up 3 months follow-up	0.287 0.357	0.212 0.203	1.831 3.090	−0.129 −0.041	0.703 0.756	0.079 0.176
	Level of education						
	Lower education^a^						
	Higher education	0.216	0.187	1.335	−0.150	0.582	0.248
	Age of youngest child	0.071	0.048	2.146	−0.024	0.165	0.143
	Total knowledge score	0.223	0.012	327.178	0.199	0.248	<0.001
	Trial groups x time point						
	Control x baseline^a^						
	Intervention x immediate follow-up Intervention x 3 months follow-up	0.538 0.286	0.235 0.230	5.254 1.548	0.078 −0.164	0.998 0.736	0.022 0.143
Perceived	Trial group						
Barriers^e^	Control^a^						
	Intervention	0.289	0.314	0.849	−0.326	0.905	0.357
	Timepoint						
	Baseline^a^						
	Immediate follow-up 3 months follow-up	1.043 0.820	0.184 0.176	32.135 21.604	0.682 0.474	1.404 1.165	<0.001 <0.001
	Level of education						
	Lower education^a^						
	Higher education	0.312	0.244	1.632	−0.167	0.790	0.201
	Total knowledge score	−0.003	0.013	0.053	−0.029	0.023	0.819
	Trial groups x time point						
	Control x baseline^a^						
	Intervention x immediate follow-up Intervention x 3 months follow-up	0.413 0.455	0.271 0.260	2.324 3.067	−0.118 −0.054	0.944 0.964	0.127 0.080
Preschool	Trial group						
Children's	Control^a^						
Handwashing	Intervention	0.072	0.166	0.189	−0.253	0.398	0.664
Practice^f^	Timepoint						
	Baseline^a^						
	Immediate follow-up 3 months follow-up	0.359 0.344	0.069 0.072	26.776 22.982	0.223 0.204	0.495 0.485	<0.001 <0.001
	Parents' total practice	0.016	0.008	4.022	0.000	0.031	0.045
	Trial groups x time point						
	Control x baseline^a^						
	Intervention x immediate follow-up	0.118	0.108	1.194	−0.094	0.330	0.275
	Intervention x 3 months follow-up	0.121	0.115	1.107	−0.104	0.347	0.293

a *Reference groups*.

b *Intercept B coefficient of 4.615*.

c *Intercept B coefficient of 7.846*.

d *Intercept B coefficient of 3.597*.

e *Intercept B coefficient of 10.840*.

f *Intercept B coefficient of 13.274*.

**Figure 3:**
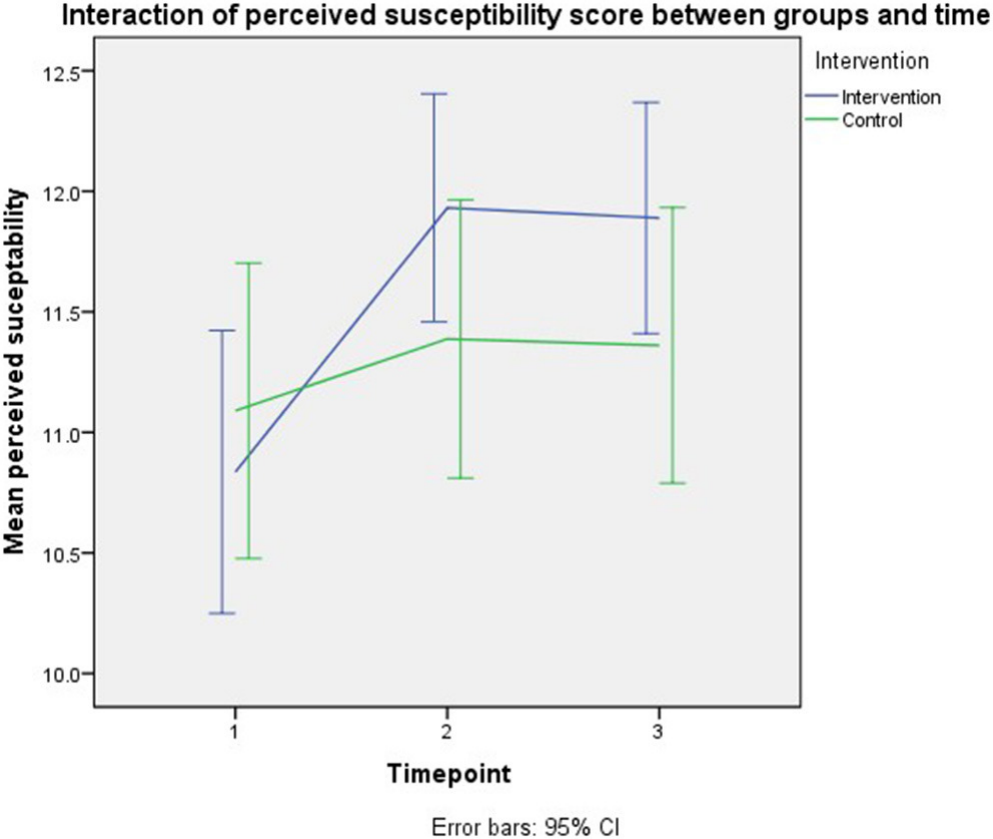
Interaction of perceived susceptibility scores between groups and time.

Meanwhile, the lower perceived susceptibility can be observed by an increment of 1 unit of the youngest child's age (*B* = −0.116, 95% CI: −0.224–−0.009, *p* = 0.034). The parents who had 1-unit higher knowledge had 0.2-unit higher perceived susceptibility (*B* = 0.245, 95% CI: 0.223–0.268, *p* < 0.001). Even though there was no direct intervention effect, the parents in the intervention group and followed up immediately the following intervention recorded higher perceived susceptibility than those in the control group (*B* = 0.510, 95% CI: 0.062–0.957, *p* = 0.026). However, no significant difference in the perceived susceptibility score at the third-month follow-up between the trial groups. Per-protocol analysis revealed the same significant model effects as the intention to treat analysis. Figure 3 demonstrates the interaction of perceived susceptibility scores between groups and time.

#### Perceived Severity

**Figure 4:**
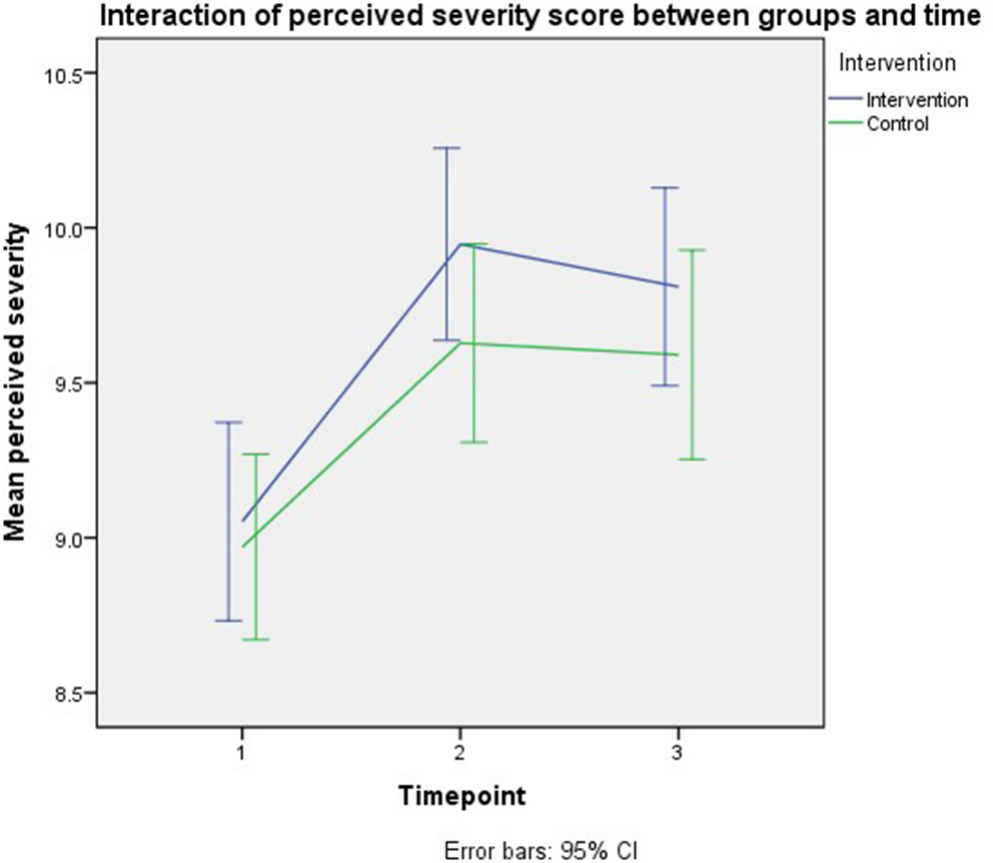
Interaction of perceived severity score between groups and time.

The smallest Quasi Likelihood under Independence Model Criterion (QIC) for the final model was 2,554. Table 2 shows the timepoint was the only significant model effect. Parents who were followed- up immediately after the intervention had higher perceived severity compared to baseline by 0.6 units (*B* = 0.603, 95% CI: 0.299–0.906, *p* < 0.001), and those who were at third-month post-intervention recorded increment in parents' perceived severity by 0.5 unit (*B* = 0.575, 95% CI: 0.264–0.887, *p* < 0.001) compared to baseline. However, no significant intervention effect was observed on the outcome. Per-protocol analysis revealed a similar model effect as the intention to treat analysis. Figure 4 depicts the interaction of perceived severity score between groups and time.

#### Perceived Benefits

**Figure 5:**
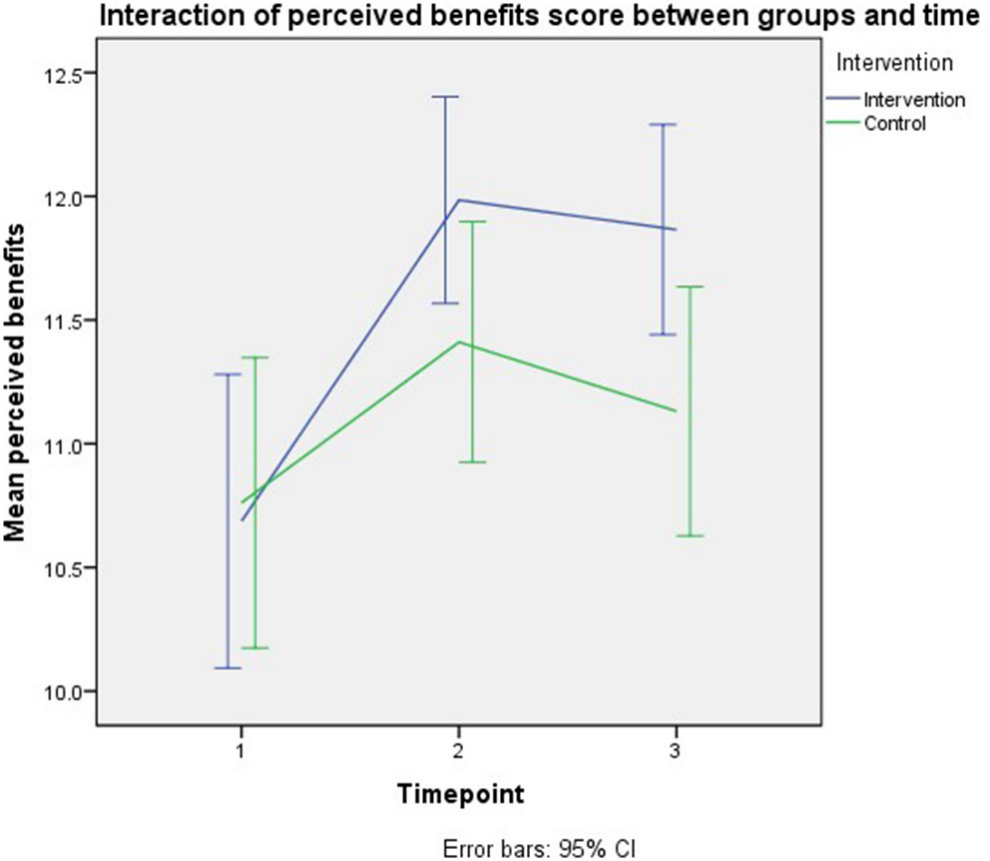
Interaction of perceived benefits scores across groups and time.

The final model's smallest Quasi Likelihood under Independence Model Criterion (QIC) was 2,454. Table 2 demonstrates that the total knowledge score and interaction term of trial groups and time point were the significant model effects for the outcome of the perceived benefit. Those with a 1-unit higher knowledge score had demonstrated higher perceived benefits (*B* = 0.223, 95% CI: 0.199–0.248, *p* < 0.001). Even though there was no significant direct intervention effect on the perceived benefits, the parents in the intervention group demonstrated a higher score at immediate follow-up than their counterparts (*B* = 0.538, 95% CI: 0.078–0.998 *p* = 0.022). However, no significant difference was observed between trial groups at third-month follow-up. Per-protocol analysis revealed similar model effects as the intention to treat analysis. Figure 5 shows the interaction of perceived benefits scores across groups and time.

#### Perceived Barriers

**Figure 6:**
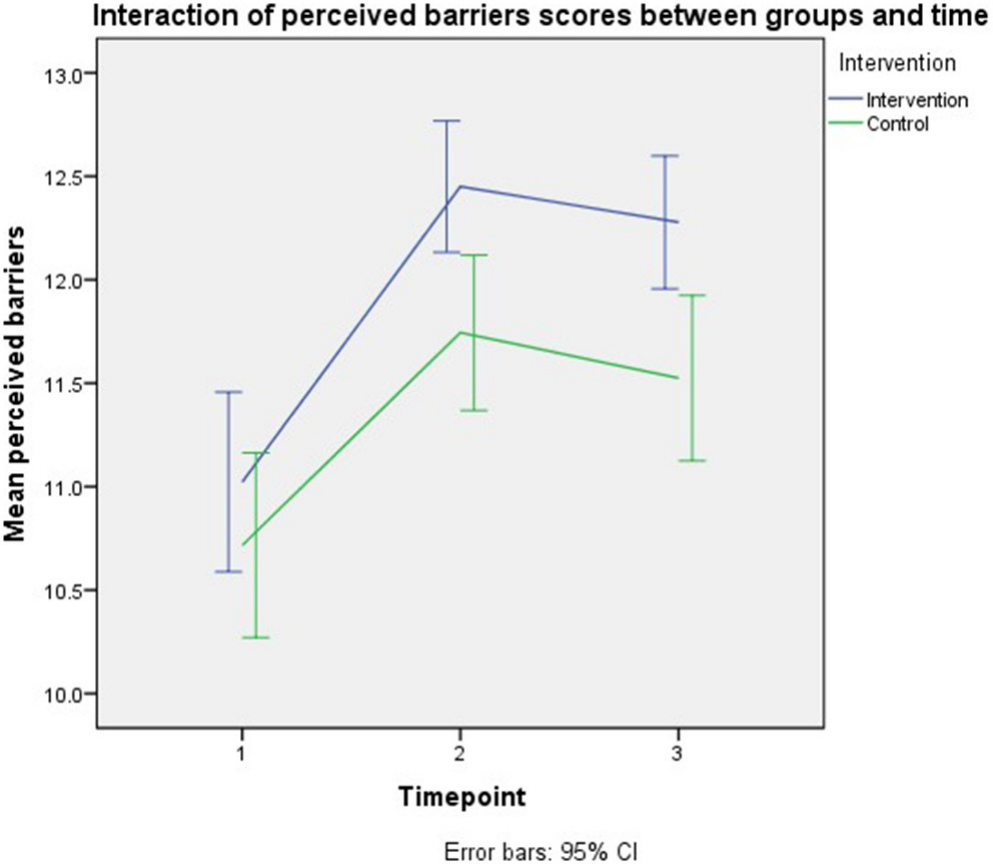
Interaction of perceived barriers scores between groups and time.

The final model's smallest Quasi Likelihood under Independence Model Criterion (QIC) was 3,830. The model in Table 2 shows that the timepoint was the only significant model effect of the perceived barriers outcome. Parents who were followed up immediately (*B* = 1.043, 95% CI: 0.682–1.404, *p* < 0.001) and after 3 months post-intervention (*B* = 0.820, 95% CI: 0.474–1.165, *p* < 0.001) showed higher perceived barriers scores than baseline, respectively. No significant intervention effect was observed on the outcome. Similar model effects were demonstrated in the per-protocol analysis, as shown in the appendix. Figure 6 shows the interaction of perceived barriers scores between groups and time.

### Preschool Children's Handwashing Practice

**Figure 7:**
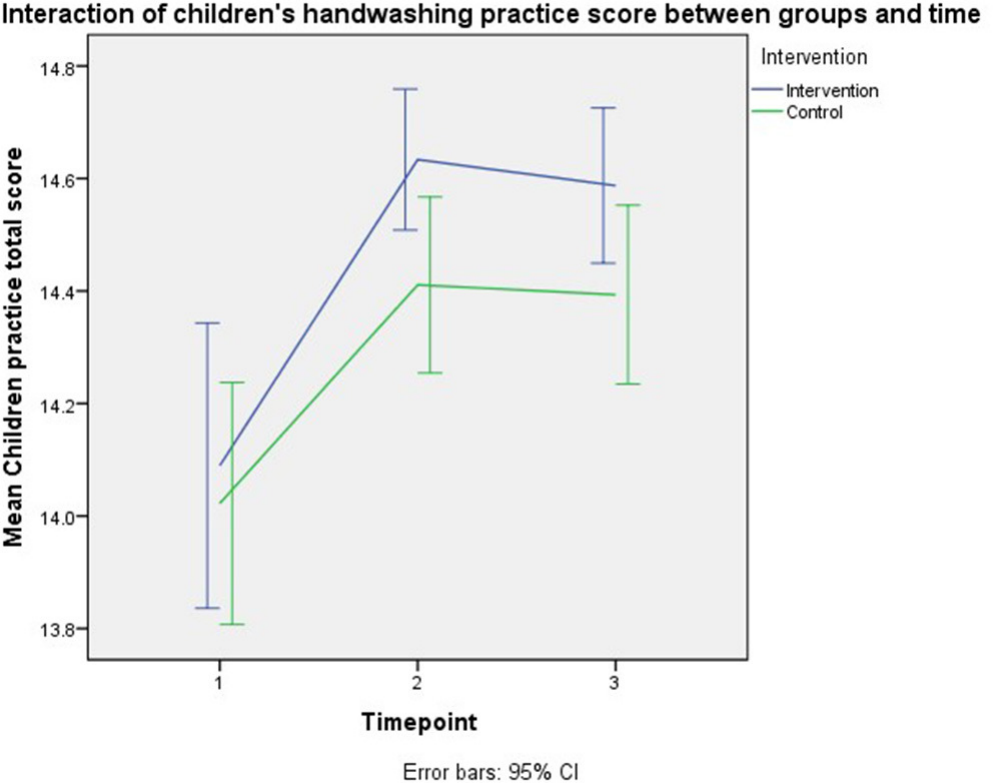
Interaction of children's handwashing practice score between groups and time.

Table 2 shows the intention to treat analysis to study the module's effectiveness on preschool children's hand hygiene practice. There was no intervention effect on the construct. However, significant model effects included different follow-up times and parents' total practice scores. Those who were follow-up at immediate post-intervention recorded higher preschool children's practice score by 0.4 unit (*B* = 0.359, 95% CI: 0.223–0.495, *p* < 0.001), whilst the score was higher at third month follow- up by 0.3 unit compared to baseline (*B* = 0.344, 95% CI: 0.204–0.485, *p* < 0.001). There was a 0.02-unit higher preschool children's hand hygiene practice score for each unit increment in parents' practice score (*B* = 0.016, 95% CI: 0.000–0.031, *p* = 0.045). However, no significant intervention effect can be observed on the outcome. Figure 7 shows the interaction of children's handwashing practice score between groups and time.

## Discussion

### Baseline Comparison of Sociodemographic and Household Characteristics

The baseline data were not much different from a study conducted in Klang Valley examining the predictors of preventive behaviors toward Hand, Foot and Mouth Disease (HFMD) among *Jabatan Kemajuan Masyarakat* (KEMAS) preschoolers mothers (17). The study reported the mean age of respondents of 35.6 (SD: 5.57), married (*N* = 340, 96.3%), attained at least secondary education (*N* = 228, 64.8%), and lived with three children in the same household (Median = 3, IQR = 2).

Similarly, another cross-sectional study on knowledge, attitude and practice of hand hygiene among preschool children's parents also reported that most of the respondents were mothers (*N* = 110, 45.8%), aged 31 to 40 years old (*N* = 135, 56.3%), attained secondary education (*N* = 155, 64.6%) and had three or more children living in the same household (*N* = 139, 56.8%) (10).

Moreover, concerning the intervention study, a multi-faceted community trial in China on HFMD and hand hygiene reported the majority of the parents attained at least secondary education in the intervention group, and it was not significantly different between groups (8). This study did not examine any other items comparable to our study except for parents' education level.

Furthermore, this study was partially comparable at baseline as most variables were similar between intervention and control groups except parents' age, age of the youngest child, location of residence and presence of active smokers. A cluster-randomized controlled study (RCT) is expected to have a significant baseline imbalance, especially in a study involving small clusters (18). Bolzern et al. (19) suggested that the baseline imbalance in a cluster RCT is related to the recruitment process and not the randomization process, usually caused by a recruitment bias whereby the respondents are recruited after randomization. However, our study did not have a recruitment bias, as the participants were recruited before randomization.

According to previous studies, parents' age was a significant covariate of parents' knowledge of hand hygiene (20, 21). Mansur and Ahmad (20) reported parents aged 46 to 50 years old had more favorable knowledge compared to younger parents (*p* < 0.001). Otherwise, the age of the youngest child, location of residence and presence of active smokers were associated with HFMD incidence, but not the outcomes of this study.

Studies suggested restricted randomization such as stratification and minimization techniques could be used in the design phase to avoid baseline imbalance (22). However, this study did not use restricted randomization techniques to avoid clusters with no participants due to the small clusters and high numbers of strata. Another suggested method is to control the covariates with a significant imbalance which had a significant association with the outcomes in multivariate analysis (22). In this study's case of knowledge outcome, the parents' age was not included in the multivariate analysis. It was not significantly associated with respondents' knowledge in our study and did not significantly change the model goodness of fit, QIC.

### Baseline Comparison of Parents' Perceived Susceptibility, Severity, Benefits and Barriers, and Preschool Children's Hand Hygiene Practice

A similar result by cross-sectional study in Thailand reported the highest score in the perceived susceptibility subscale (*N* = 373, 31.8%) and the lowest score in the perceived severity subscale (*N* = 343, 75.2%) (21). A survey in Klang among mothers of preschool children reported the highest score in the perceived susceptibility subscale (Median = 13, IQR = 2), and the perceived barriers were the subscale with the lowest score (Mean = 7.80, SD 1.24) (17). Perceived susceptibility was the construct with the highest scores in all studies, most probably contributed by the high knowledge score on the causative agents and mode of transmission of HFMD.

On the other hand, the lower perceived severity score was contributed by the knowledge on the complications of HFMD. Our trial reported an excellent knowledge level on the HFMD complications, thus, affecting the perceived severity scores. Most respondents thought that the infection was very severe and needed hospitalization in most cases. The finding is consistent with Rosenstock (23), who postulated that a person may perceive a health problem regarding its clinical consequences and whether it could lead to death. Besides, the frequent media reporting on HFMD in Malaysia, especially during the massive epidemic in 2018, makes people perceive HFMD as a severe disease. The idea is parallel with the findings of an experimental study among psychology undergraduate students, which reported that the diseases reported frequently by the media were often perceived as more severe than those reported less commonly (24).

### Baseline Comparison of Preschool Children's Handwashing Practice

Similarly, a randomized controlled community trial in China also revealed good baseline handwashing habits of children (Mean = 11.2, SD 5.6 in intervention group vs. Mean = 11.3, SD 4.8 in control group, *p* = 0.446) (8). Meantime, a multi-center intervention study in Klang among 435 preschoolers estimated that most children washed their hands before and after meals (*N* = 361, 83%) and after out from the toilet (*N* = 288, 66.2%), but not after playing outdoor (*N* = 213, 49%) and after sneezing or coughing (*N* = 173, 39.8%) (25). Unlike our study, this trial reported self-reporting measures on handwashing practice from the preschool children themselves, supported by observation on handwashing techniques.

### The Effectiveness of the Module

This study had significant intervention effects on parents' perceived susceptibility and benefits outcomes at immediate follow-up, but not at the third-month follow-up. According to the graph of the interaction effect of trial groups and time, all outcome scores were initially increased at immediate follow-up. Still, later the scores were slightly reduced at the third-month follow-up leading to the non-significant intervention effect of all the outcomes at third-month follow-up compared to baseline, except on the parents' knowledge. Such findings were postulated due to the nature of our intervention, which was conducted only at a one-time point rather than conducted repeatedly, making the scores higher at immediate follow-up than third-month follow-up.

In contrast, a community trial of hand hygiene and Hand Foot and Mouth Disease (HFMD) among parents of young children in China, the parents' knowledge [β (year 1) = 0.194, 95% CI: 0.100–0.288 vs. β (year 2) = 0.191, 95% CI: 0.097–0.284], and handwashing habits [β (year 1) = −1.831, 95% CI: 1.933–−1.788 vs. β (year 2) = −1.818, 95% CI: −1.920–−1.716] as well as children's handwashing habits [β (year 1) = −1.847, 95% CI: −1.950–−1.745 vs. β (year 2) = −1.876, 95% CI: −1.979–−1.773], were significantly different at year 1 and year 2 of intervention (8). Compared to our trial, the trial in China was a huge trial involving 32 villages at each arm. It also involved intensive health education, including training of trainers among local doctors compared to only general health education given to the control group. It was conducted at two HFMD epidemic sessions (April 1–July 31) in two consecutive years: 2011 and 2012.

Another cluster randomized controlled trial on hand hygiene in India also showed a further significant increment of the outcome as the trial progressed (26). At six weeks' follow-up, handwashing with soap at key events was more common in the intervention group than in the control group [19% (*SD* = 21) vs. 4% (*SD* = 2), *p* = 0·005]. At the 6-month follow-up visit, the proportion of handwashing with soap was even higher; 37% (*SD* = 7) in the intervention group vs. 6% (*SD* = 3) in the control group (*p* = 0·02). Compared to our study, this trial assessed a different aspect of hand hygiene practice; the proportion of handwashing at key events, rather than the frequency of handwashing among children or the parents' preventive practice against HFMD, including hand hygiene as in our study. Furthermore, the handwashing practice of parents and children were clustered together as a common handwashing outcome among household members. Thus, the handwashing practice on different respondents could not be determined. Besides, this study was a vast, funded trial, which involved 14 villages with 700 to 2,000 villagers for each village. It also involved a one-month intensive intervention in the intervention group, while the control group received a delayed, shorter version of intervention (9 days). Furthermore, the content of the intervention was multi-faceted, as it involved many activities such as flip chart presentation, filmmaking, house-to-house visits by promoters, broadcasting of campaign songs, pledging among community members, school events and community events, tallied to the local community circumstances, to cater for various types of respondents in the study.

### Effectiveness of the Module on Parents' Perceived Susceptibility, Severity, Benefits and Barriers

This trial showed that timepoints were the significant predictors of the perceived susceptibility, severity, and barriers but not the perceived benefits. Besides, knowledge was also significantly affected perceived susceptibility and perceived benefits in this study. Even though there were no intervention effects on all outcomes, the perceived susceptibility and perceived benefits showed significant increment at immediate follow-up in the intervention group compared to the control group at baseline.

The findings can be explained by the readily high scores at the baseline, especially on the perceived susceptibility outcome (Mean = 10.84, SD = 3.43 in the intervention arm and Mean = 11.09, SD = 3.59 in the control arm), which may produce ceiling effects, in which the apparent treatment effect might be lowered due to the high baseline scores. Thus, only the intervention effect will be significant after adding the time point. Similarly, the perceived barriers also showed a higher baseline score in the intervention group, in which out of a total score of 15, the baseline mean score was 11.02 (SD = 2.54) for the intervention group compared to only mean score of 10.72 (2.61) for the control group which may lead to the insignificant treatment effect due to higher baseline score in the intervention group at the first place.

There is scarce literature on hand hygiene interventions, which assessed the Health Belief Model (HBM) constructs as part of the outcomes. A quasi-experimental study among 135 nurses in Iran, involving four sessions of health education focusing on nosocomial infection, reported a significant intervention effect on the perceived threats (*p* = 0.004), perceived benefits related to the nurses (*p* < 0.001) and patients (*p* < 0.001), and perceived barriers related to the nurses (*p* < 0.001) and hospital (*p* < 0.001) (13). This study was conducted in 2 army hospitals, and four sessions of health education lasted for 45 min for each session were conducted at different shifts. The assessments using the self-made questionnaires were done at the baseline and two months post-intervention.

### Effectiveness of the Module on Preschool Children's Handwashing Practice

Our study showed no intervention effect on preschool children's handwashing practice, but a significant difference in children's practice at different follow-up times and different parents' practice scores. In contrast, the community trial in China involving parents of 6 to 40-months old children revealed intervention effects on the preschool children's handwashing practice (*B* = 1.847, *p* < 0.001) (8).

As explained before, the contradicting finding is mainly due to the different intervention modalities and length of time. Besides, the readily high score of children's handwashing practice at baseline in our study also may contribute to the non-significant difference in the score between intervention groups, as the difference was too little to be noticed. In contrast, the moderate baseline score of children's handwashing practice in the China trial still had room for improvement via intervention.

## Conclusion

In conclusion, when compared to baseline, our intervention improved parents' perceived susceptibility and benefits outcomes. Unfortunately, no significant intervention effects were found on parents' perceptions of severity and barriers, as well as preschool children's handwashing practices. The length of time between follow-ups significantly impacted each outcome. In addition to intervention groups and follow-up time, significant covariates were outcome predictors in our study. Apart from parents' knowledge and perceived susceptibility, the age of the youngest child was a significant predictor of parents' perceived susceptibility.

### Limitation

The timing of this trial during the COVID-19 pandemic influenced the effectiveness of our intervention. Due to the pandemic COVID-19 and the concern of virus transmission among community members, the data collecting measures were changed from face-to-face intervention to online intervention package distribution. Furthermore, the high absenteeism rate of preschool children due to fear of COVID-19 and government enforcement of Movement Control Order (MCO) made it challenging to investigate more objective outcomes like HFMD incidence or absenteeism rate, as reported by most trials on hand hygiene.

An attitude change in behaviors is essential in many investigations (10, 21, 26, 27). However, this solution did not address the module's attitude because motivating people via an online platform was difficult. The intervention's one-time nature may contribute to the non-significant intervention impact at the third month. The effects may fade with time. Face validity conducted among graduate students may not represent our study respondents' educational background, as most of our study participants completed primary and secondary school. As a result, the questionnaires may be interpreted differently. In addition, due to time constraints, the researchers only included parents, who the author believes are the most important predictors of children's health, particularly in the COVID-19 pandemic condition. This study did not include teachers because of time and resource restrictions despite the importance of teaching children about HFMD and basic handwashing practices.

This study also relied on parents' self-reporting data. For example, appraising their own children's handwashing habits may be skewed. This study's high baseline handwashing rate suggests that parents may have overestimated their children's scores.

In our study, the control group got the hand hygiene and HFMD brochure as part of the intervention package. Due to the pandemic, several media outlets have already promoted hand hygiene messages. The goal is to compare the efficiency of various health promotion strategies. Thus, this study evaluated the effectiveness of an intervention that employed verbal, visual, and written communication to one that used written communication. However, this diluted the intervention impact. The intervention group's parents' perceived severity and barriers did not change, nor did their children's hand hygiene practices. Most outcomes improved over time in both study arms showed that both health communication approaches could improve outcomes.

As a result of the COVID-19 pandemic, our results may not generalize to clusters (preschools) or individual participants (parents of preschool children) as the baseline findings and intervention effectiveness differed. For example, a different time with less information on hand hygiene might have lowered the baseline. However, the intervention impact would have been better if the trial had not occurred during the COVID-19 pandemic.

### Recommendations

Another study suggests that attitudes and motivation are crucial in modifying behavior. Thus, future research may include attitudes as an objective. If finances allow, future interventions could be repeated to maintain behavioral change and achieve desired goals. Research should use a similar background sample to provide unbiased feedback and assessment. If time permits, the correctness and thoroughness of parents' and preschoolers' handwashing procedures can be assessed. Then include the preschool teachers and children as participants. Future studies can use objective measures to supplement respondents' self-reported data. Common quantitative hand hygiene metrics include HFMD incidence, illness absence rates, and surface bacteria colony counts (8, 28–31). The module can be implemented in numerous ways (using multiple health education strategies) to suit different participants. Having hand soap or other hand hygiene facilities in the module can be a plus if resources allow. It could be used as a cue to action in the Health Belief Model (HBM) to remind participants to wash their hands. Despite being undertaken during the COVID-19 pandemic, the hand hygiene and HFMD modules showed good participation rates and positive outcomes. As a result, the program will be expanded to other schools with a high risk of infection.

## Data Availability Statement

The raw data supporting the conclusions of this article will be made available by the authors, without undue reservation.

## Ethics Statement

The approval to conduct this study was obtained from the Human Ethics Committee of Universiti Putra Malaysia (UPM). This study was registered under clinicaltrials.gov as per procedure (Trial number: TCTR20200211003). The patients/participants provided their written informed consent to participate in this study.

## Author Contributions

HS, SM, and KM: performed conceptualization, validation, writing review, editing, visualization, and manuscript supervision. SS: performed methodology, formal analysis, investigation, and original draft writing. All authors have read and approved the manuscript.

## Conflict of Interest

The authors declare that the research was conducted in the absence of any commercial or financial relationships that could be construed as a potential conflict of interest.

## Publisher's Note

All claims expressed in this article are solely those of the authors and do not necessarily represent those of their affiliated organizations, or those of the publisher, the editors and the reviewers. Any product that may be evaluated in this article, or claim that may be made by its manufacturer, is not guaranteed or endorsed by the publisher.

## References

[1] Koh WM Badaruddin H La H Chen MI-C Cook AR. Severity and burden of hand, foot and mouth disease in Asia: a modelling study. BMJ Glob Health. (2018) 3:e000442. 10.1136/bmjgh-2017-000442 29564154 PMC5859810

[2] Salleh NM Agus Salim NA Kamaruzzaman SN Mahyuddin N Darus FM. The prevelence of SBS and absenteeism among children in urban refurbished private preshools. MATEC Web Conf. (2016) 66:00119. 10.1051/matecconf/20166600119

[3] Zou X Zhang X Wang B Qiu Y. Etiologic and epidemiologic analysis of hand, foot, and mouth disease in Guangzhou city: a review of 4,753 cases. Braz J Infect Dis. (2012) 16:457–65. 10.1016/j.bjid.2012.08.001 22964289

[4] Wei Ang L Koh BK Peng Chan K Bact D Tee Chua L James L. Epidemiology and control of hand, foot and mouth disease in Singapore, 2001-2007. Ann Acad Med Singap. (2009) 108:106–12. 19271036

[5] Aldinger CE Whitman CV. Case Studies in Global School Health Promotion. New York, NY: Springer New York (2009).

[6] Klein M Chong P. Is a multivalent hand, foot, and mouth disease vaccine feasible? Hum Vaccin Immunother. (2015) 11:2688–704. 10.1080/21645515.2015.1049780 26009802 PMC4685682

[7] Wang J Hu T Sun D Ding S Carr MJ Xing W. Epidemiological characteristics of hand, foot, and mouth disease in Shandong, China, 2009–2016. Sci Rep. (2017) 7:8900. 10.1038/s41598-017-09196-z 28827733 PMC5567189

[8] Guo N Ma H Deng J Ma Y Huang L Guo R. Effect of hand washing and personal hygiene on hand food mouth disease. Medicine (Baltimore). (2018) 97:e13144. 10.1097/MD.0000000000013144 30572426 PMC6320109

[9] Liu X Hou W Zhao Z Cheng J van Beeck EF Peng X. A hand hygiene intervention to decrease hand, foot and mouth disease and absence due to sickness among kindergarteners in China: a cluster-randomized controlled trial. J Infect. (2019) 78:19–26. 10.1016/j.jinf.2018.08.009 30134143

[10] Mohamed NA Zulkifli Amin NN Ramli S Mohamed Salleh N Isahak I. Knowledge, attitudes and practices of hand hygiene among parents of preschool children. J Sci Innov Res. (2016) 5:1–6.34720505

[11] May B. Adult HFMD Cases Increasing in United States. Available online at: https://www.contagionlive.com/view/adult-hfmd-cases-increasing-in-united-states (accessed August 11, 2021).

[12] Yin X Yi H Shu J Wang X Wu X Yu L. Clinical and epidemiological characteristics of adult hand, foot, and mouth disease in northern Zhejiang, China, May 2008 – November 2013. BMC Infect Dis. (2014) 14:251. 10.1186/1471-2334-14-251 24885052 PMC4026826

[13] Zeigheimat F Ebadi A Rahmati-Najarkolaei F Ghadamgahi F. An investigation into the effect of health belief model-based education on healthcare behaviors of nursing staff in controlling nosocomial infections. J Educ Health Promot. (2016) 5:23. 10.4103/2277-9531.184549 27500176 PMC4960766

[14] Shin Y Kim E Shin H Lee J Jeong S. A program to build early school-aged child's personal hygiene habits based on health belief model. Korean J Heal Promot. (2018) 18:51. 10.15384/kjhp.2018.18.1.51

[15] Lwanga SK Lemeshow S. Sample Size Determination In Health Status : A Practical Manual, 1st Edn. WHO (1991). p. 77.

[16] Correa JC Pinto D Salas LA Camacho JC Rondón M Quintero J. cluster-randomized controlled trial of handrubs for prevention of infectious diseases among children in Colombia. Rev Panam Salud Pública. (2012) 31:476–84. 10.1590/S1020-49892012000600005 22858814

[17] Suliman Q Said SM Afiah N Zulkefli M. Predictors of preventive practices towards HFMD among mothers of preschool children in Klang District. Malaysian J Med Heal Sci. (2017) 13:21–32.

[18] Carter B. Cluster size variability and imbalance in cluster randomized controlled trials. Stat Med. (2010) 29:2984–93. 10.1002/sim.4050 20963749

[19] Bolzern JE Mitchell A Torgerson DJ. Baseline testing in cluster randomized controlled trials: should this be done? BMC Med Res Methodol. (2019) 19:1–5. 10.1186/s12874-019-0750-8 31101078 PMC6524320

[20] Mansur NNH Ahmad A. Knowledge and prevention practices of hand, foot and mouth disease among parents and caregivers in Bandar Puncak Alam, Selangor, Malaysia. Malaysian J Public Health Med. (2021) 21:29–36. 10.37268/mjphm/vol.21/no.1/art.485

[21] Ruttiya Charoenchokpanit TP. Knowledge attitude and preventive behaviors towards hand foot and mouth disease among caregivers of children under five years old in Bangkok, Thailand. J Heal Res. (2013) 27:281−6.

[22] Wright N Ivers N Eldridge S Taljaard M Bremner S. A review of the use of covariates in cluster randomized trials uncovers marked discrepancies between guidance and practice. J Clin Epidemiol. (2015) 68:603–9. 10.1016/j.jclinepi.2014.12.006 25648791 PMC4425474

[23] Rosenstock IM. The health belief model and preventive health behavior. Heal Educ Behav. (1977) 2:354–86. 10.1177/109019817400200405

[24] Young ME Norman GR Humphreys KR. Medicine in the popular press: the influence of the media on perceptions of disease. PLoS ONE. (2008) 3:3552. 10.1371/journal.pone.0003552 18958167 PMC2569209

[25] Tengku Jamaluddin TZM Mohamed NA Mohd Rani MD Ismail Z Ramli S Faroque H. Assessment on hand hygiene knowledge and practices among preschool children in Klang Valley. Glob Pediatr Health. (2020) 7. 10.1177/2333794X20976369 33335950 PMC7724414

[26] Biran A Schmidt WP Varadharajan KS Rajaraman D Kumar R Greenland K. Effect of a behaviour-change intervention on handwashing with soap in India (SuperAmma): a cluster-randomized trial. Lancet Glob Heal. (2014) 2:e145–54. 10.1016/S2214-109X(13)70160-8 25102847

[27] Eshetu D Kifle T Hirigo AT. Knowledge, attitudes, and practices of hand washing among Aderash primary schoolchildren in Yirgalem Town, Southern Ethiopia. J Multidiscip Healthc. (2020) 13:759–68. 10.2147/JMDH.S257034 32821113 PMC7423343

[28] Rosen L Manor O Engelhard D Brody D Rosen B Peleg H. Can a handwashing intervention make a difference? Results from a randomized controlled trial in Jerusalem preschools. Prev Med (Baltimore). (2006) 42:27–32. 10.1016/j.ypmed.2005.09.012 16300823

[29] Willmott M Nicholson A Busse H MacArthur GJ Brookes S Campbell R. Effectiveness of hand hygiene interventions in reducing illness absence among children in educational settings: a systematic review and meta-analysis. Arch Dis Child. (2016) 101:42–50. 10.1136/archdischild-2015-308875 26471110 PMC4717429

[30] Sandora TJ Shih M-C Goldmann DA. Reducing absenteeism from gastrointestinal and respiratory illness in elementary school students: a randomized, controlled trial of an infection-control intervention. Pediatrics. (2008) 121:e1555–62. 10.1542/peds.2007-2597 18519460

[31] Lennell A Kühlmann-Berenzon S Geli P Hedin K Petersson C Cars O. Alcohol-based hand-disinfection reduced children's absence from Swedish day care centers. Acta Paediatr Int J Paediatr. (2008) 97:1672–80. 10.1111/j.1651-2227.2008.01057.x 18945282

